# The first complete chloroplast genome of *Cosmos sulphureus* Cav. 1791 (Asteraceae) and its phylogenetic analysis

**DOI:** 10.1080/23802359.2026.2648173

**Published:** 2026-03-30

**Authors:** Qingxia Chen, Ruolan Gao, Chang Liu, Youjie Yao

**Affiliations:** a The First Clinical School of Zhengzhou University, Zhengzhou University, Zhengzhou City, PR China; b Department of Cardiology, The First Affiliated Hospital of Zhengzhou University, Zhengzhou City, PR China

**Keywords:** Asteraceae, comparative genomics, *Cosmos sulphureus*, medicinal plant, phylogenetic analysis

## Abstract

*Cosmos sulphureus* is an annual ornamental plant with pharmaceutical and biopesticidal value. Here, we report its first complete chloroplast genome (150,870 bp), featuring a typical quadripartite structure and 37.52% GC content. The genome comprised 129 predicted genes, including 86 protein-coding genes, 35 tRNAs, and eight rRNAs. A triple inversion was identified in the LSC region: an ∼83.9 kb large repeat nested an ∼18.8 kb medium repeat, which in turn enclosed a ∼3.4 kb small repeat. Phylogenetically, *C. sulphureus* and *C. bipinnatus* form a monophyletic clade, diverging ∼1.593 Mya. These findings provide genetic resources for future studies of the genus.

## Introduction

*Cosmos sulphureus* Cav. 1791, commonly known as yellow cosmos, is an annual ornamental plant in the Asteraceae family, native to Mexico and widely distributed across tropical and subtropical regions of the Americas (Lim 2013). Valued for its vibrant yellow to orange flowers, it is used both in cuisine—such as in salads and teas—and in traditional Mexican medicine for the treatment of stomatitis and scorpion stings (Kaisoon et al. 2012; Ortega-Medrano et al. 2023).

Phytochemical investigations have identified diverse bioactive compounds in its leaf and flower extracts, including phenolics, flavonoids, tannins, and terpenoids (de Morais et al. 2020). Specific constituents such as chlorogenic acid, caffeic acid, rutin, and quercetin contribute to notable antioxidant activity, highlighting its potential pharmaceutical and biopesticidal applications (Saleem et al. 2019; Irsyadi et al. 2022).

Anatomically, *C. sulphureus* exhibits characteristic Asteraceae features such as dorsiventral leaves and anomocytic stomata (Tahir et al. 2017). Additionally, *C. sulphureus* has been examined in phylogenetic studies to clarify its relationships within the genera *Cosmos*, *Bidens*, and *Dahlia*. It is closely related to *Cosmos atrosanguineus*, as demonstrated by plastid subtype identity sequencing, which has facilitated successful interspecific hybridization (Oku et al. 2008).

Despite these advances in phytochemical and morphological characterization, genomic resources for *C. sulphureus* remain limited. In particular, no chloroplast genome has been reported to date. Here, we present the first complete chloroplast genome of *C. sulphureus* to support future phylogenetic and genetic research in *Cosmos* and related genera.

## Materials and methods

**Figure 1.**
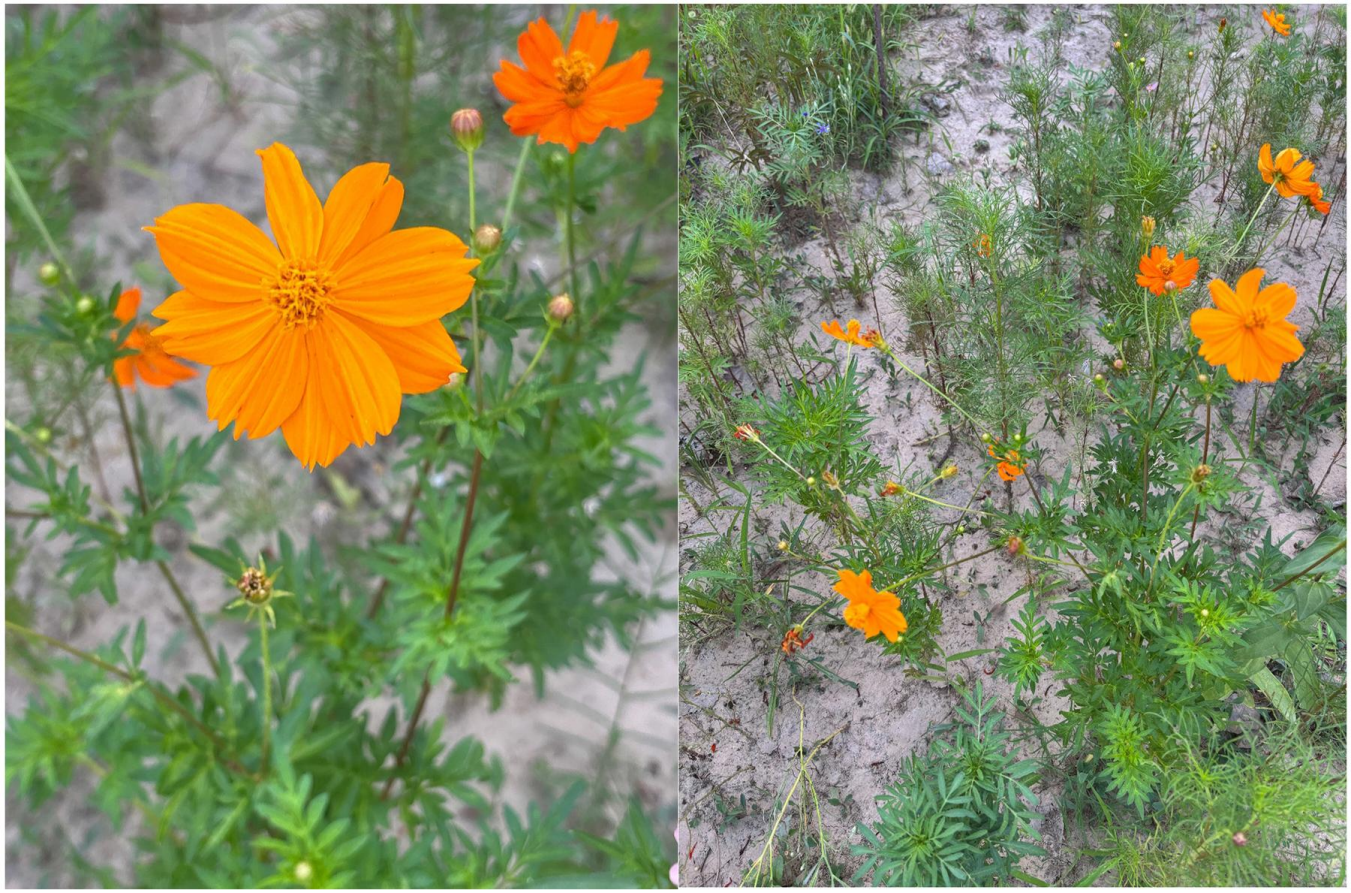
Field photographs of *C. sulphureus* showing habit and diagnostic features (photographer: Qingxia Chen). The plant can reach a height of 1.5–2 m and is pubescent. The leaves are bipinnate with lanceolate lobes. The capitulum measures approximately 2.5–5 cm in diameter, consisting of orange or golden-yellow ray florets, which can be either single or double and possess three apical teeth, and yellow disk florets. The achenes are brownish.

Healthy fresh leaves of *C. sulphureus* used for sequencing were collected from Zhengzhou University, Zhengzhou City, Henan Province, China (113°38′24″E, 34°44′43″N) (Figure 1). The voucher specimen was deposited in the Herbarium of Zhengzhou University (contact: Qingxia Chen, 13675308787@163.com) under the accession number ZZU-2025-0839.

To ensure high-quality DNA extraction, the CTAB method (Doyle and Doyle 1987) was applied. DNA integrity was assessed on a 1% agarose gel, and purity was verified by OD260/280 ratios between 2.0 and 2.2 measured using a NanoDrop2000 spectrophotometer (Thermo Scientific, Waltham, MA). DNA concentration was quantified with a Qubit 3.0 fluorometer. For library construction, 300 ng to 1 µg of high-quality DNA was enzymatically fragmented to an average size of 200–400 bp. Sequencing was conducted on the DNBSEQ platform with 150 bp paired-end reads. Raw reads were filtered using Fastp (v0.21.0; Chen et al. 2018) to obtain clean reads according to the following criteria: removal of reads containing >5% ambiguous bases (N), >50% low-quality bases (quality ≤5), adapter contamination, or PCR duplicates.

The complete chloroplast genome was assembled using GetOrganelle (v1.7.1) (Jin et al. 2020) with parameters ‘-R 50 -k 21,45,65,85,105 -P 1000000 -F embplant_pt’. Annotation was performed in CPGAVAS2 (Shi et al. 2019) using the ‘43-plastomes’ reference set and manually validated in Apollo (v1.11.6) (Pontius 2018). The genome was visualized with Chloroplot (Zheng et al. 2020), and sequencing depth and coverage were plotted using DrawSeqDepth (https://github.com/wlqg1983/DrawSeqDepth) under default settings.

To determine the phylogenetic position of *C. sulphureus*, 21 chloroplast genomes from Coreopsideae (Asteraceae) were analyzed, including 18 newly retrieved sequences. The dataset included two *Cosmos* species (excluding *C. sulphureus*), two *Dahlia*, one *Coreopsis* (two samples), 13 *Bidens*, and two outgroups (*Gaillardia pulchella* and *Marshallia obovata*). A total of 73 shared protein-coding genes were extracted and aligned using MAFFT (v7.505; Katoh and Standley 2016). Maximum-likelihood phylogeny was reconstructed using IQ-TREE (v2.2.2.7; Nguyen et al. 2015) under the TVM + F + I + R4 model, with nodal support evaluated through 1000 bootstrap replicates. Divergence times were estimated via Bayesian inference (BI) of the 73-gene alignment using BEAST (v10.5.0; Suchard et al. 2018), applying a strict clock and the GTR substitution model. The MCMC analysis ran for 1,000,000 generations, sampling every 1000 generations under a constant coalescent tree prior. Calibration employed a 6.74-million-year divergence time for the two outgroups (Kumar et al. 2022). Tracer (v1.6; http://tree.bio.ed.ac.uk/software/tracer) confirmed effective sample sizes >200. After a 10% burn-in, TreeAnnotator was used to generate the maximum clade credibility tree, which was visualized in FigTree (v. 1.4.3; http://tree.bio.ed.ac.uk/software/figtree/).

## Results

**Figure 2.**
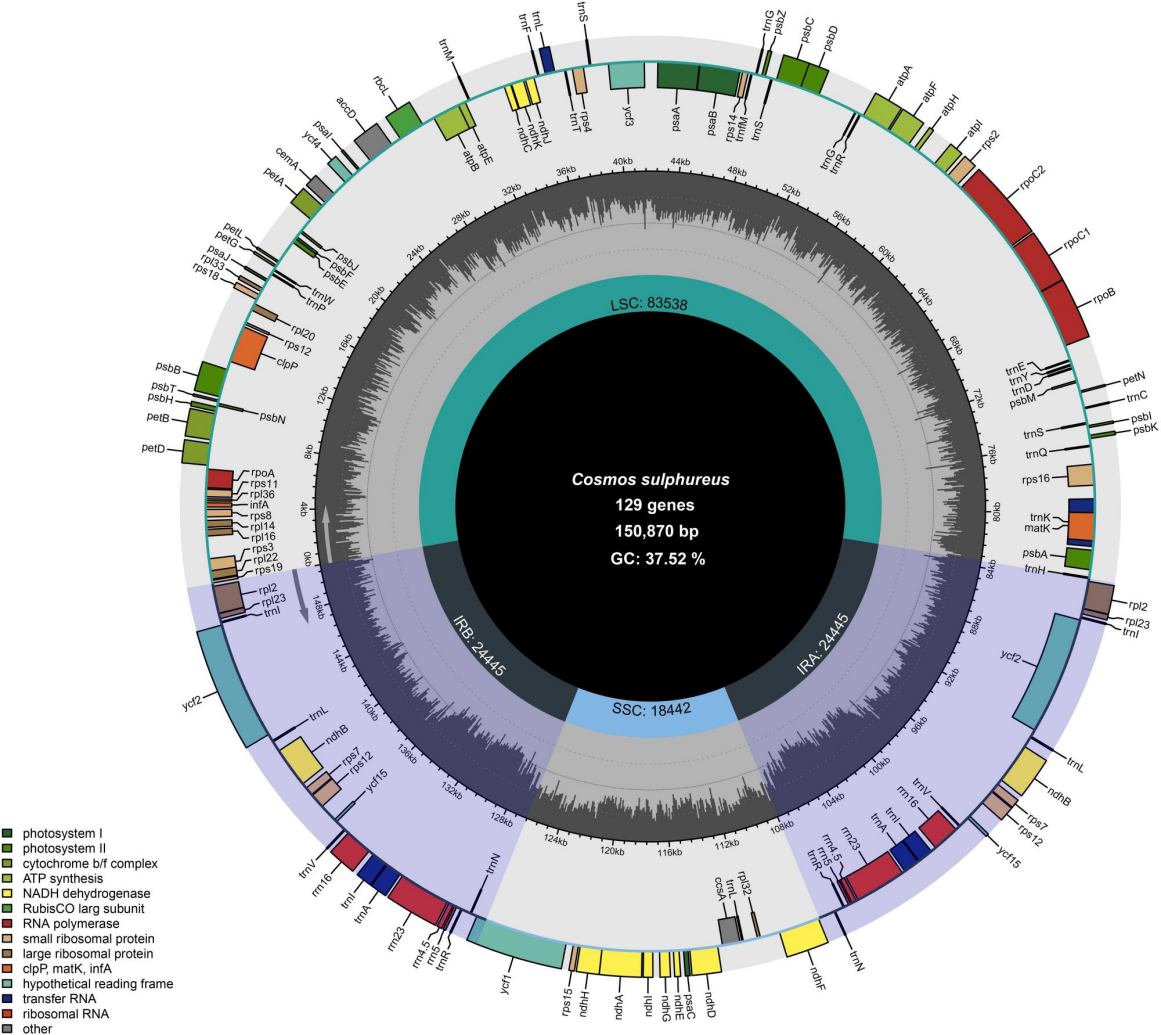
The chloroplast genome map of *C. sulphureus*. The chloroplast genome displayed the typical quadripartite structure consisting of a large single-copy (LSC) region, a small single-copy (SSC) region, and a pair of inverted repeats (IRa and IRb). Genes were color-coded by functional category and were distributed on both strands of the genome. The inner gray ring indicated the GC content across the genome. Functional categories included genes involved in photosystem I and II, cytochrome b6/f complex, ATP synthase, NADH dehydrogenase, RNA polymerase, ribosomal proteins (small and large subunits), Rubisco, tRNAs, rRNAs, and hypothetical chloroplast reading frames (*ycf*).

After sequencing, quality filtering and preprocessing, at least 20 Gb of clean whole-genome sequencing data (fastq format) were obtained. Based on these clean data, a high-quality chloroplast genome of *C. sulphureus* was assembled. The chloroplast genome measured 150,870 bp in length and displayed a typical quadripartite structure (Figure 2). In the mapping analysis, the chloroplast genome exhibited an average sequencing depth of 1001.62×, with a minimum depth of 131× and a maximum depth of 2075× (Figure S1). The genome comprised two inverted repeat (IR) regions, each 24,445 bp in length, separated by a large single-copy (LSC) region of 83,538 bp and a small single-copy (SSC) region of 18,442 bp (Figure 2).

The chloroplast genome exhibited a heterogeneous GC content distribution, with an overall GC content of 37.52%. The highest GC content was observed in the IR regions (43.10%), whereas the corresponding values were 35.68% and 31.02% for the LSC and SSC regions, respectively. Compared with the chloroplast genome of *Barnadesia caryophylla* (OM892817.1, Barnadesioideae, Asteraceae), a triple IR region was identified in the LSC of the *C. sulphureus* chloroplast genome, in which a large ∼83.9 kb inversion contained a nested medium-length ∼18.8 kb inversion, and within this medium-length inverted region, a small ∼3.4 kb inversion further nested (Figure S2).

The annotated chloroplast genome of *C. sulphureus* contained 129 genes (107 unique genes) (Table S1), comprising 86 protein-coding genes (79 unique), eight rRNA genes (four unique), and 35 tRNA genes (26 unique). Additionally, the genome contained 18 (16 unique) cis-splicing genes (11 protein-coding genes and seven tRNA genes) and one trans-splicing gene (*rps*12). The schematic maps of the cis-splicing and trans-splicing PCGs are shown in Figures S3 and S4. The trans-splicing gene *rps*12 had two 3′ tails, each containing one intron in the two IR regions. Among the cis-splicing genes, 16 genes (*atp*F, *rpo*C1, *rps*16, *rpl*2 (×2), *ndh*B (×2), *ndh*A, *ycf*1, *trn*L-UAA, *trn*G-GCC, *trn*K-UUU, *trn*I-CAU, *trn*I-GAU, *trn*A-UGC (×2)) had one intron and two genes (*clp*P, and *ycf*3) had two introns.

**Figure 3.**
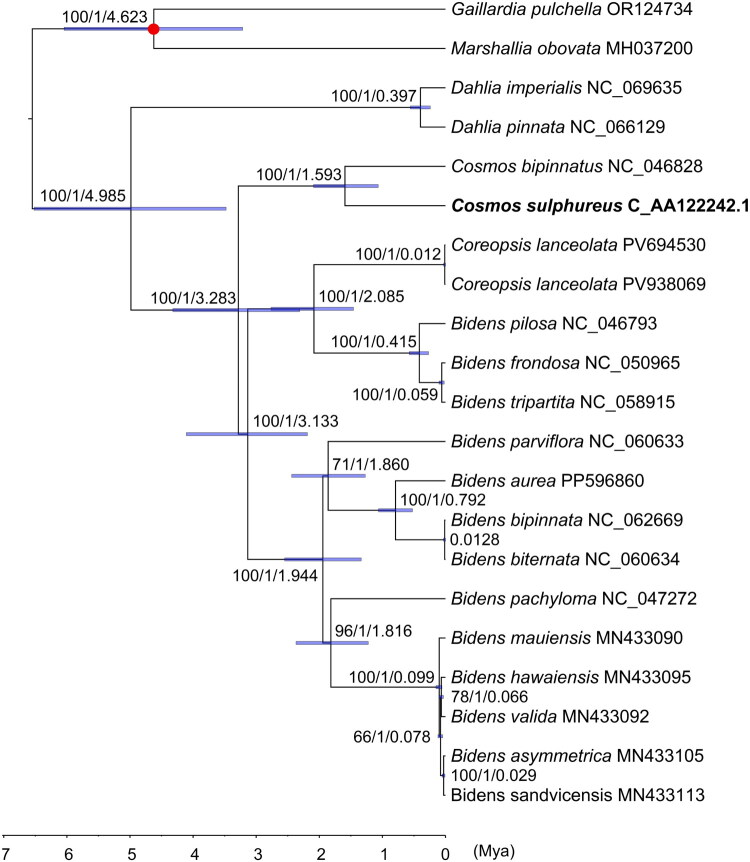
ML and BI phylogeny of *C. sulphureus* and its close relatives. Bootstrap support values (ML) and posterior probabilities (BI) were shown at each node, along with estimated divergence times (TMRCA). Blue bars represented the 95% highest posterior density (HPD) intervals for divergence times inferred from the BI analysis. The red dot indicated the calibration point used for molecular dating. The detailed chloroplast genome citation information was: *Dahlia imperialis* (NC_069635), *D. pinnata* (NC_066129), *C. bipinnatus* (NC_046828) (Jiang et al. 2019), *C. sulphureus* (C_AA122242.1, a newly reported chloroplast genome deposited in CNCB, labeled by bold font), *Bidens frondosa* (NC_050965) (Li et al. 2020), *B. tripartita* (NC_058915) (Wu et al. 2022), *B. pilosa* (NC_046793) (Lin et al. 2020), *Coreopsis lanceolata* (PV694530), *Coreopsis lanceolata* (PV938069) (Wan et al. 2025), *B. aurea* (PP596860) (Mao et al. 2024), *B. bipinnata* (NC_062669) (Zhang et al. 2023), *B. biternata* (NC_060634) (Wu et al. 2022), *B. parviflora* (NC_060633) (Wu et al. 2022), *B. pachyloma* (NC_047272) (Knope et al. 2020), *B. mauiensis* (MN433090) (Knope et al. 2020), *B. hawaiensis* (MN433095) (Knope et al. 2020), *B.* valida (MN433092) (Knope et al. 2020), *B. asymmetrica* (MN433105) (Knope et al. 2020), *B. sandvicensis* (MN433113) (Knope et al. 2020), *Gaillardia pulchella* (outgroup, OR124734) (Li et al. 2025), and *Marshallia obovata* (outgroup, MH037200).

In the phylogenetic analysis of *C. sulphureus*, based on BI and ML methods, the topologies of the two trees were identical. The results indicated that *C. sulphureus* and *C. bipinnatus* formed a monophyletic group with 100% bootstrap support, while *Cosmos*, *Bidens*, and *Coreopsis* together constituted a broader monophyletic clade. Additionally, *Dahlia* and *Cosmos* species formed separate monophyletic groups (Figure 3). The divergence times of the *Dahlia* and *Cosmos* monophyletic groups were estimated at ∼0.397 Mya and ∼1.593 Mya, respectively. The *Bidens* species were inferred to be polyphyletic, having diverged about ∼3.133 Mya ago. This robust phylogenetic reconstruction confirms the taxonomic position of *C. sulphureus* within Coreopsideae of Asteraceae.

## Discussion and conclusions

We report the first chloroplast genome of *C. sulphureus* and describe its primary characteristics, which include a typical quadripartite structure with a size of 150,870 bp and 129 predicted genes. Phylogenetic analysis indicated that *C. sulphureus* clustered into a monophyletic group with *C. bipinnatus*.

Compared with the double inversion commonly observed in Asteraceae chloroplast genomes (Salih et al. 2017), *C. sulphureus* exhibits a more complex triple inversion in the LSC hotspot, likely generated through successive recombination events (Walker et al. 2014). These findings underscore that complete chloroplast genome sequencing, rather than limited markers, is essential for identifying such complex structural variations and for more reliable interpretation of evolutionary relationships (Kim et al. 2005).

From a taxonomic perspective, *C. sulphureus* has been investigated in phylogenetic studies to clarify its relationships within the genera *Cosmos*, *Bidens*, and *Dahlia*. Oku et al. (2008) used plastid subtype identity (PSID) sequences to resolve the phylogenetic position of the closely related chocolate cosmos (*C. atrosanguineus*), confirming its closer affinity to *Cosmos* than to *Bidens* or *Dahlia*. In this study, *C. sulphureus* clustered into a monophyletic group with *C. bipinnatus*, consistent with the results of Oku et al. (2008). The genus *Bidens* is polyphyletic, whereas Coreopsis is paraphyletic, which agrees with the findings of Knope et al. (2020). *Bidens* diverged about ∼3.133 Mya, similar to the estimated divergence time of ∼3.14 Mya for the most recent common ancestor of Pacific and American lineages (Knope et al. 2020). In summary, the reported chloroplast genome of *C. sulphureus* can support future research on species identification, genetic diversity, and phylogenetic evolution.

## Supplementary Material

Figure_S4.jpg

Figure_S1.jpg

Figure_S2.jpg

Table_S1.docx

Figure_S3.jpg

## Data Availability

The complete chloroplast genome sequence of *C. sulphureus* in this study was submitted to CNCB-NGDC at https://ngdc.cncb.ac.cn/genbase/ under the accession number C_AA122242.1. The associated BioProject, Run Accession, and BioSample numbers for raw sequencing data in CNCB-NGDC (https://ngdc.cncb.ac.cn/gsa/) were PRJCA047390, CRR2188722, and SAMC5967396, respectively.
