# Supplementary figures and images for: The first complete chloroplast genome of Cosmos sulphureus Cav. 1791 (Asteraceae) and its phylogenetic analysis
Source: Mitochondrial DNA B Resour. 2026 Mar 30;11(5):572–6. doi: 10.1080/23802359.2026.2648173 (PMC13037155; doi:10.1080/23802359.2026.2648173)

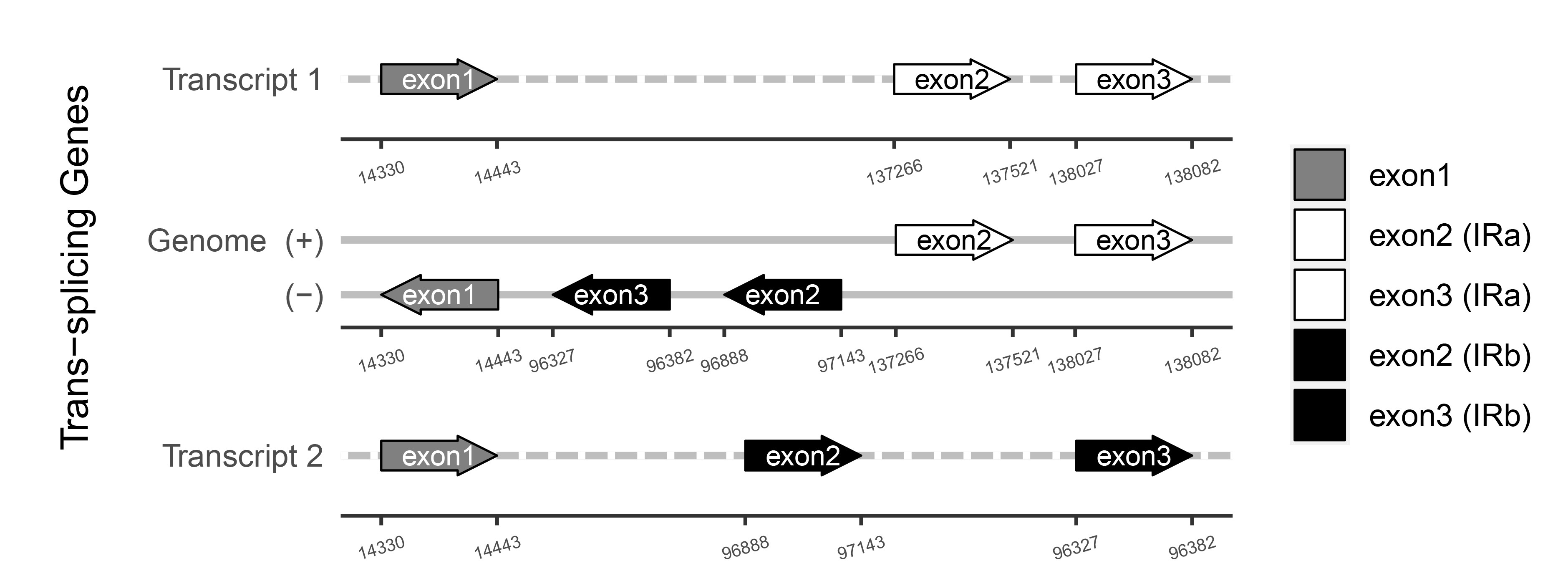

Supplement: Figure_S4.jpg [file TMDN_A_2648173_SM0078.jpg]

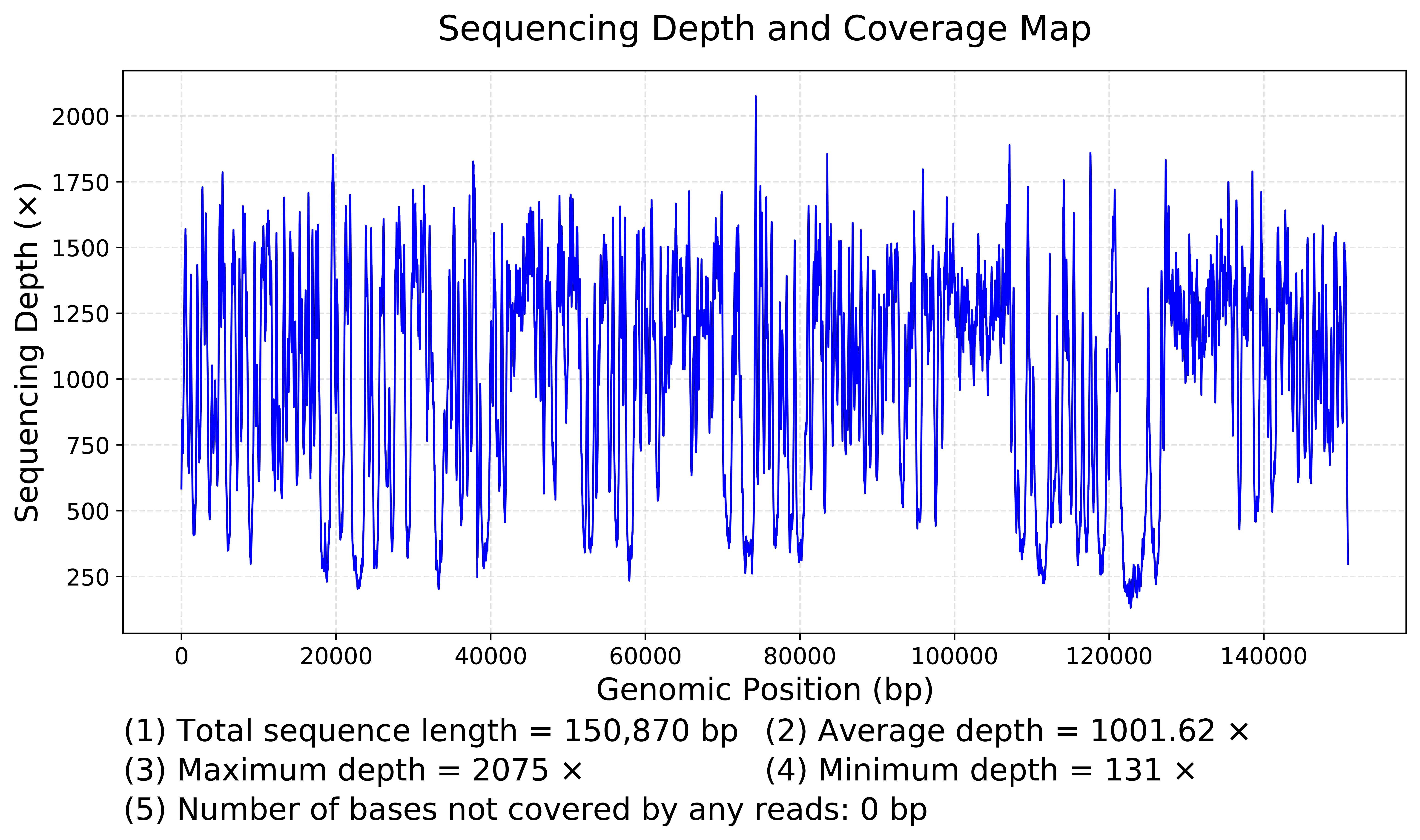

Supplement: Figure_S1.jpg [file TMDN_A_2648173_SM0077.jpg]

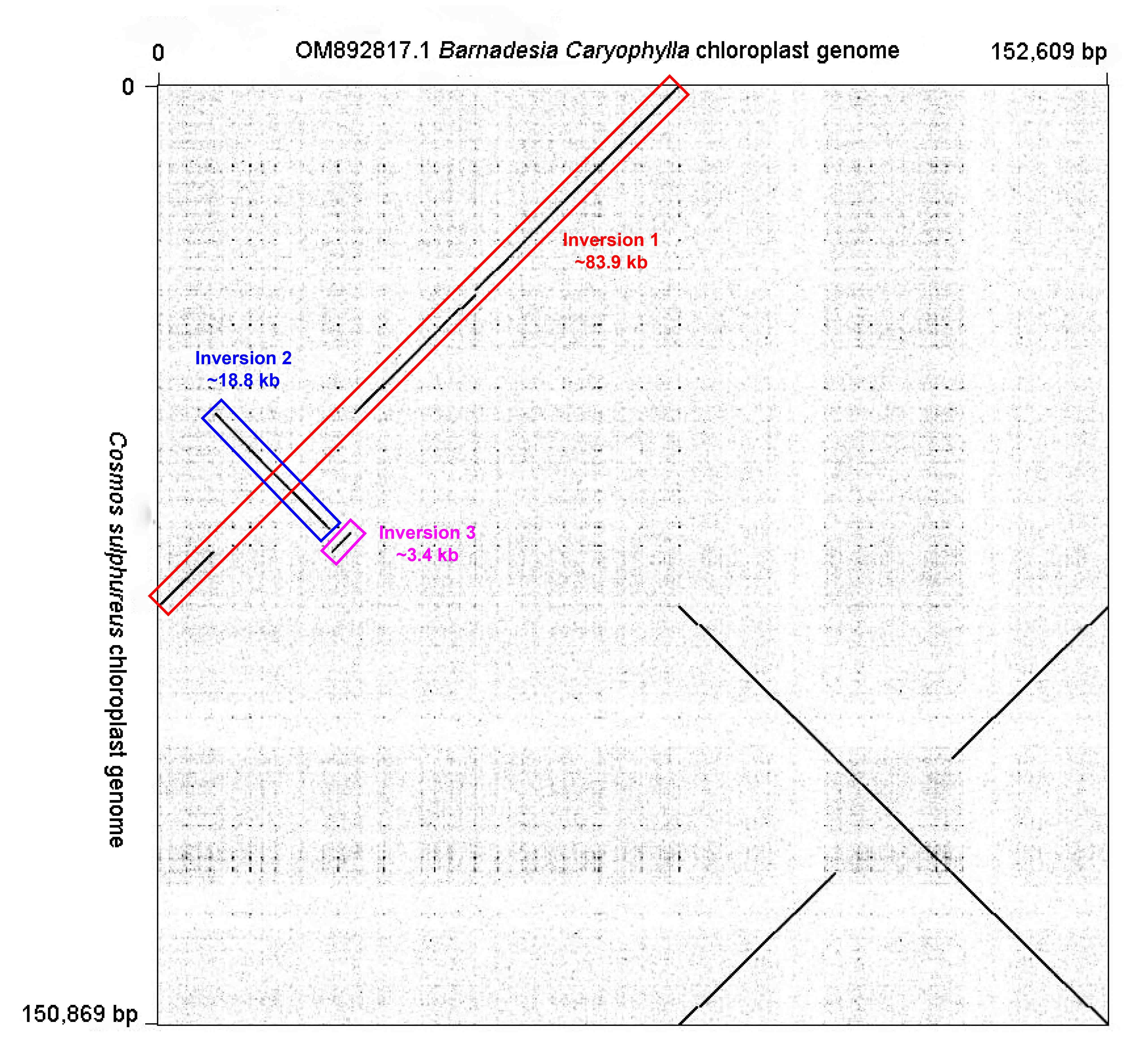

Supplement: Figure_S2.jpg [file TMDN_A_2648173_SM0076.jpg]

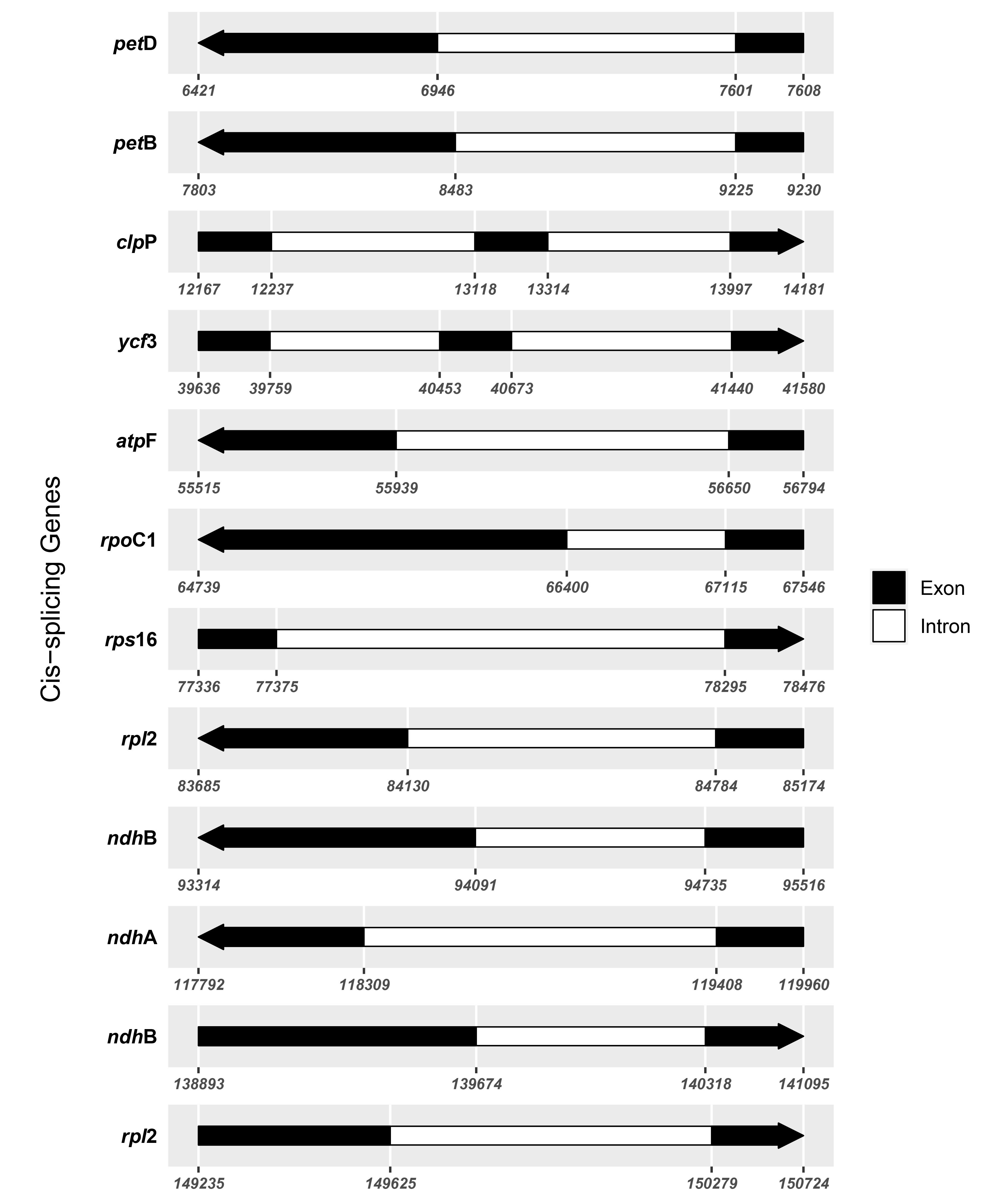

Supplement: Figure_S3.jpg [file TMDN_A_2648173_SM0074.jpg]
