# Supplementary material for: The first complete chloroplast genome of Cosmos sulphureus Cav. 1791 (Asteraceae) and its phylogenetic analysis
Source: Mitochondrial DNA B Resour. 2026 Mar 30;11(5):572–6. doi: 10.1080/23802359.2026.2648173 (PMC13037155; doi:10.1080/23802359.2026.2648173)
Supplement: Table_S1.docx [file TMDN_A_2648173_SM0075.docx]

**Table** **S1.** **Genes** **annotated** **in** **the** ***C. sulphureus*** **chloroplast** **genome.**

| **Gene** **function** | **Gene** **type** | **Gene** **name** |
| --- | --- | --- |
| rRNA | rRNA genes | *rrn*4.5S(×2), rrn5S(×2), *rrn*16S(×2), *rrn*23S(×2) |
| tRNA | tRNA genes | *trn*P-UGG, *trn*W-CCA, *trn*fM-CAU, *trn*F-GAA, *trn*L-UAA*, *trn*T-UGU, *trn*S-GCU(×2), *trn*M-CAU, *trn*S-UGA, *trn*G-GCC*, *trn*G-GCC, *trn*R-UCU, *trn*E-UUC, *trn*Y-GUA, *trn*D-GUC, *trn*C-GCA, *trn*Q-UUG, *trn*K-UUU*, *trn*H-GUG, *trn*I-CAU(×2), *trn*I-CAU*, *trn*I-GAU*, *trn*L-CAA(×2), *trn*V-GAC(×2), *trn*A-UGC(×2)*, *trn*R-ACG(×2), *trn*N-GUU(×2), *trn*L-UAG |
| Selfduplicate | Small subunit of ribosome | *rps*2, *rps*3, *rps*4, *rps*7(×2), *rps*8, *rps*11, *rps*12(×2)*, *rps*14, *rps*15, *rps*16*, *rps*18, *rps*19 |
|  | Large subunit of ribosome | *rpl*2(×2)*, *rpl*14, *rpl*16*, *rpl*20, *rpl*22, *rpl*23(×2), *rpl*32, *rpl*33, *rpl*36 |
|  | DNA dependent RNA polymerase | *rpo*A, *rpo*B, *rpo*C1*, *rpo*C2 |
| Photosynthesis | Subunits of NADH-dehydrogenase | *ndh*A*, *ndh*B(×2)*, *ndh*C, *ndh*D, *ndh*E, *ndh*F, *ndh*G, *ndh*H, *ndh*I, *ndh*J, *ndh*K |
|  | Subunits of photosystem Ⅰ | *psa*A, *psa*B, *psa*C, *psa*I, *psa*J |
|  | Subunits of photosystem Ⅱ | *psb*A, *psb*B, *psb*C, *psb*D, *psb*E, *psb*F, *psb*H, *psb*I, *psb*J, *psb*K, *psb*M, *psb*N, *psb*T, *psb*Z, *ycf*3** |
|  | Subunits of cytochrome b/f complex | *pet*A, *pet*B*, *pet*D*, *pet*G, *pet*L, *pet*N |
|  | Subunits of ATP synthase | *atp*A, *atp*B, *atp*E, *atp*F*, *atp*H, *atp*I |
|  | Large subunit of rubisco | *rbc*L |
| Other genes | Maturase | *mat*K |
|  | Protease | *clp*P** |
|  | Envelope membrane protein | *cem*A |
|  | Subunit of Acetyl-CoA-carboxylase | *acc*D |
|  | c-type cytochrom synthesis gene | *ccs*A |
|  | Translational initiation factor | *inf*A |
| Genes of unknown functions | Open Reading Frame | *ycf*1, *ycf*2(×2), *ycf*4, *ycf*15(×2) |
| Note: Numbers in the parentheses represent the number of copies. *, **: indicte one and two introns, respectively. | | |
